# Kinetic and isotherm insights of Diclofenac removal by sludge derived hydrochar

**DOI:** 10.1038/s41598-022-05943-z

**Published:** 2022-02-09

**Authors:** Sadish Oumabady, Paul Sebastian Selvaraj, Kalaiselvi Periasamy, Davamani Veeraswamy, Paulian Thankanadathi Ramesh, Thava Palanisami, Sangeetha Piriya Ramasamy

**Affiliations:** 1 Department of Environmental Sciences, Tamil Nadu Agricultural University, Coimbatore, 641 003 India; 2 Agricultural College and Research Institute, Tamil Nadu Agricultural University, Killikulam, 628 252 India; 3 Global Innovative Centre for Advanced Nanomaterials (GICAN), The University of Newcastle, Callaghan, NSW 2308 Australia

**Keywords:** Environmental sciences, Chemistry

## Abstract

Recently, hydrothermal carbonization emerges as the most viable option for the management of solid waste with high moisture content. Sludge derived hydrochar is used as an adsorbent for emerging contaminants or micro-pollutants in the domain of sustainability. Current study demonstrates the KOH activation of hydrochar produced from paper board mill sludge and evaluates its removal potential of a Non-steroidal anti-inflammatory drug, Diclofenac from aqueous solution. The activated hydrochars exhibited porous, spherical micro-structures with higher fraction of oxygenated functional groups paving way for the efficient adsorption of Diclofenac. The effect of initial Diclofenac concentration and contact time was ascertained using adsorption kinetics and isotherms. The adsorption kinetics exhibited second-order reaction for all adsorbents indicating higher coefficient of determination **(**R^2^ > 0.9). The Diclofenac adsorption on hydrochars followed Langmuir isotherm model with the post-activated hydrochar recording a highest adsorption capacity of 37.23 mg g^−1^ in 40 mg L^−1^ initial Diclofenac concentration at 15 h equilibrium time.

## Introduction

In 2017, the worldwide paper production was 413 million tonnes, with India accounting for 3.18% of total paper, newsprint, and paperboard production per annum^1^. The paper board mill industry salvages these paper products after utilization to produce recycled materials such as corrugated cardboard, wrapper and packaging boxes. The effluent treatment plants (ETP) of these industries generates semi-solid slurry from primary and secondary clarifiers known as paper board mill sludge which are managed by landfill formations and incineration. It is a heterogeneous biomass material containing very merger solid (10%) and higher moisture content (90%)^1^. Handling and disposal of ETP sludge is a challenging task for every industry, since it possesses many regulatory issues and requires high management cost besides environmental degradation^2^. Hydrothermal carbonization (HTC) serves to overcome the energy-consumptive drying process for the management of organic feedstock with higher moisture content^3^. HTC performs in relatively mild reaction temperatures between 180 and 250 °C for 5–240 min under auto generated pressure conditions^4^. The main advantages of HTC over other thermal processes are the use of moderate temperatures, non-requirement of inert atmosphere, kills pathogens and degrades the thermally labile pollutants. The surface modified carbon product generated from HTC known as hydrochar widens the array of its applications *i.e.* energy generation and storage, adsorption of contaminants and soil amendment^5,6^. The hydrolysis and dehydration reactions during HTC promote the production of oxygenated functional groups thus making it an effective carbon material^7^. Additionally, metallic concentrations of iron and calcium get concentrated and mediate the adsorption of anionic contaminants^8,9^. An increase in carbon material’s cumulative surface area with a net negative charge can be achieved by improving coarseness and promoting crack development through surface modification methodologies^10^. This will result in the enhancement of removal potential of environmental contaminants from aqueous media.

The adsorption capacity of hydrochar can be improved by activation using chemical agents^8^. It depends on its porous nature and the kind of surface functional groups which is further influenced by the type of activation, activating agent, activation temperature and the impregnating ratio^11^. A carbon material with high surface area is produced through KOH activation in which high temperature promotes the dissociation of KOH to form K_2_O and further reduction to metallic K. This broadens the void between the carbonaceous layers thereby increasing the overall surface area^12^. In addition, the pore formation on adsorbent’s surface is augmented due to the generation of CO_2_ during simultaneous gasification. During KOH activation, functional groups like phenolic, lactone, carbonyl, hydroxyl and carboxyl are generated. The formation of oxygenated functional groups is boosted up by the facile contact of the chemical with the carbon material’s surface thereby enhancing the micro-pores and meso-pores generation^13^. Activation of hydrochar enhanced the porous nature of the material with the generation of large number of active sites available for the adsorption. In addition, porosity played a crucial part in improving the surface area of activated hydrochar. The activation temperature is directly proportional to the overall surface area and porosity, however, temperature more than 800 °C reduced the porosity due to the aggregation of the already prevailing pores^14^. In a study, the effect of temperature on the porosity of KOH activated carbon materials depicted that an increase in the hydrothermal temperature beyond 280 °C directed towards a chemically stable and structurally ordered carbon^15^. The magnitude of functional groups produced is directly proportional to the KOH to char ratio, wherein a ratio of 0.5:1 promoted enhanced adsorption potential^16^. Activated carbon with high adsorption sites and porous nature help in removing emerging contaminants like Diclofenac from polluted environments.

Diclofenac 2-[(2,6-dichlorophenyl)amino] benezene acetic acid sodium salt, an anti-inflammatory non-steroidal drug with ubiquitous source leads to significant impacts on aquatic species as it remains biologically active and it enters into food chain. It is considered to be the most utilised non-steroidal anti-inflammatory drug (NSAID) with average consumption of 0.33 ± 0.29 g person^−1^ year^−1^. The NSAIDs are consumed by around 30 million people per day in average and these are excreted into the sewer system as parent chemicals or intermediates due to inadequate metabolization^17^. These chemicals have a potential threat to the environment even at low concentration of ng L^−1^ or μg L^−1^. Globally, Diclofenac is consumed in huge amount (1443 ± 58 tons) per year^18^. The maximum concentrations of Diclofenac at the global level were detected in drinking water (56 ng L^−1^), surface water (57.1 μg L^−1^), ground water (13.4 μg L^−1^), sea water (10.2 μg L^−1^), wastewater (836 μg L^−1^), soil (μg Kg^−1^), sediment (309 ng g^−1^), suspended solid (1.3 μg g^−1^), sludge (4968 μg Kg^−1^), leachate (108 μg L^−1^), fish (11.9 μg L^−1^), mussel (4.5 μg Kg^−1^) and plants (11.6 μg Kg^−1^)^19^. The occurrence of 580 unique Pharmaceuticals and Personal Care Products (PPCP) in different matrices of the environment were recently screened using a downsized database of 133 studies. It was noticed that the frequency of Diclofenac was one among the top five most commonly detected PPCPs in various matrices^20^. Furthermore, Diclofenac contributed to a 95% reduction in the vulture population in the Indian subcontinent during the 1990s owing to its renal failure and also poses serious threat to other animals, plants and aquatic organisms^21^. A study conducted in the wastewater treatment plant sites in India reported that the total NSAIDs recorded were between 0.02 and 30 mg day^−1^ per 1000 individual^22^. Moreover, a recent research on the removal of Diclofenac using activated carbon was performed at the initial sorbate concentration of 24 to 218 mg L^−1^ and reported the maximum adsorption capacity to be 180 mg g^−1^^23^.

**Figure 1:**
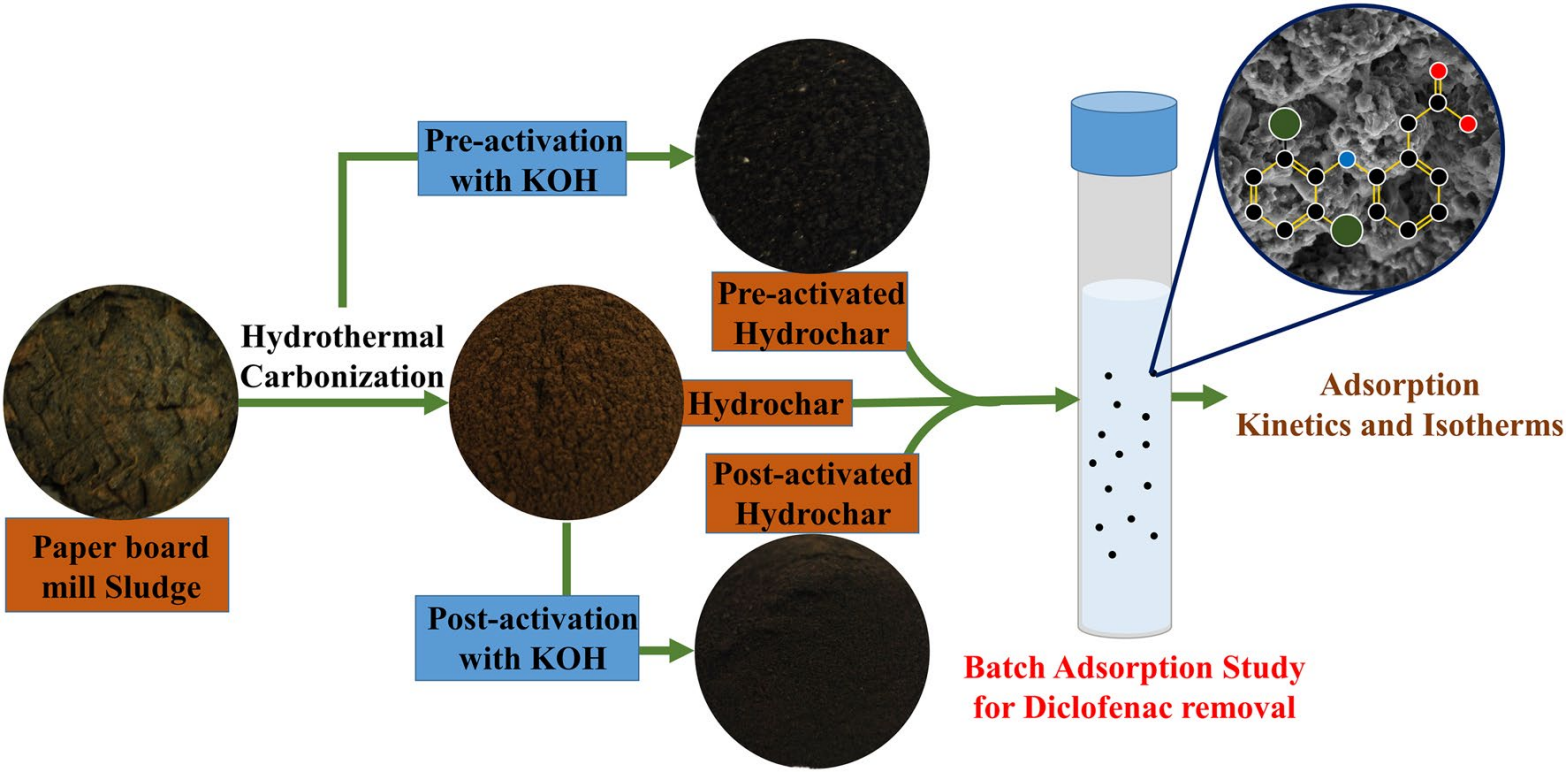
Graphical abstract of the current study.

The Diclofenac removal from the aquatic environment is generally performed through conventional methods like advanced oxidation, ozonation, membrane filtration, electrochemical oxidation, ion exchange, photocatalysis and adsorption among which the latter was found to be more effective in terms of cost and removal efficiency^24^. So, it is important to synthesize/produce low-cost adsorbent carbon materials. Most of the studies involved in the removal of Diclofenac through adsorption utilizes biodegradable non-hazardous raw material, but the present investigation attempts to utilize paper board mill sludge as a substrate for adsorbent preparation. The management of sludge is a major problem for the industries due to its hazardous nature and the cost of its disposal. With this background, the manuscript illustrates the KOH activation of hydrochar produced from paper board mill ETP sludge. Subsequently, the raw hydrochar and activated hydrochars were used for the removal of Diclofenac from synthetic aqueous solution using batch adsorption studies (Fig. 1).

## Experimental methods

This study is a continued part of our earlier published research involving the production of hydrochar from paper board mill sludge and utilizing its activated forms for the removal of orthophosphate from aqueous solutions^1,25^.

### Hydrochar production

Effluent treatment plant of ITC Ltd., PSPD (Kovai unit), Coimbatore, India was the sample collection site for the paper board mill sludge. The samples were stored in containers at 4 °C and HTC was performed in a hydrothermal autoclave reactor at 200 °C for 10 h^1^. The produced hydrochar (HC) was oven-dried to constant weight, grounded and preserved for adsorption studies.

#### KOH pre-activation and post-activation

The main objective of performing pre- and post-activation of hydrochar is to compare the characteristic differences and their adsorption capacities. The pre-activation was performed by mixing potassium hydroxide pellets with paper board mill sludge at KOH to sludge ratio of 2:1 followed by HTC at 200 °C for 10 h. In post activation, the hydrochar was mixed with potassium hydroxide pellets at KOH to char ratio of 2:1 and the mixture was kept in tubular furnace at 600 °C for 1 h in steel tubular reactor under N_2_ atmosphere with heating rate of 5 °C min^−1^. The activated hydrochar was leached with 5 M HCl to remove excess KOH and given a wash with deionized water. The pre-activated hydrochar (PRHC) and post-activated hydrochar (POHC) were oven-dried, grounded and preserved for adsorption studies^26,27^.

### Hydrochar characterization

The N_2_ adsorption/desorption behavior of hydrochars were determined at 77 K using Belsorp mini II analyzer. The adsorbents were degassed in vacuum at 110 °C and kept overnight followed by further analysis^28^. Smartsorb 92/93 surface area analyzer was used to assess the BET surface area of hydrochars. FTIR (Shimadzu’s 8400S model) was used to detect the molecular functionalities prevailing in the hydrochar at 400–4000 cm^−1^ wavenumber range^29^. Horiba Scientific Nanopartica SZ-100 particle size analyzer was used to analyze the zeta potential of hydrochars^30^. FEI—Quanta 250 Scanning Electron Microscope (SEM) was used to examine the structural morphologies of hydrochars at a voltage of 8 kV with × 10,000 magnification. FEI—Quanta 250 Transmission Electron Microscope (TEM) was used to interpret the hydrochar’s internal morphologies at an operating voltage 120 kV^31^. The point of zero charge (pH_zpc_) was determined using pH drift method.

### Batch adsorption studies

The batch adsorption studies were performed in 25 ml of different Diclofenac concentration (10, 20, 30, 40 and 50 mg L^−1^) at distinct time intervals (30, 60, 180, 360, 540, 720, 900 and 1080 min) using 0.4 g L^−1^ of raw (HC), Pre-activated (PRHC) and Post-activated Hydrochar (POHC) in neutral pH. Orbital shaker was used to provide continuous agitation to the experimental setup at 50 rpm. The removal efficiency and uptake per gram of adsorbents were calculated using the following Eq. (1) and (2).

1 $$\text{Removal\,efficiency }\left(\text{\%}\right)= \frac{({\text{C}}_{0}-{\text{C}}_{\text{e}})}{{\text{C}}_{0}}\times 100$$

2 $$\text{Amount \,of\, Diclofenac \,adsorbed \,by \,hydrochar}, {\text{Q}}_{\text{e}} (\text{mg }{\text{g}}^{-1})= \frac{({\text{C}}_{0}-{\text{C}}_{\text{e}})}{\text{m}}\times \text{V}$$

where, C_0_ is the initial Diclofenac concentration (mg L^−1^), C_e_ is the equilibrium Diclofenac concentration (mg L^−1^), m is the adsorbent dry weight (g) and V is the volume of water.

Adsorption isotherms were derived for different Diclofenac concentration ranging between 10 and 50 mg L^−1^ at the appropriate equilibrium time in continuation to the kinetic studies. The pseudo-first-order kinetic model is stated by Eq. (3), where the amount of Diclofenac adsorbed (mg g^−1^) at equilibrium and at time t (min) is given by Q_e_ and Q_t_, respectively and k_1_ is Lagergren rate constant (min^−1^)^32^. The pseudo second order kinetic model is exhibited by the Eq. (4), Where k_2_ is the second order rate constant (min^−1^)^32^. The intra particle diffusion model is expressed by the Eq. (5), with an intercept C and intra particle diffusion rate constant k_i_ (mg g^−1^ h^−1/2^). In the elovich model Eq. (6), α is the rate of initial adsorption (mg g^−1^ min^−1^) and δ is the surface coverage extent (g mg^−1^).

3 $$\text{log} \left({Q}_{e}-{Q}_{t}\right)=log{Q}_{e}- \frac{{k}_{1}}{2.303} t$$

4 $$\frac{t}{{q}_{t}}= \frac{1}{{k}_{2}Q{e}^{2}}+ \frac{t}{{Q}_{e}}$$

5 $${Q}_{t}= {k}_{i}.{t}^{1/2 }+C$$

6 $$\frac{d{Q}_{t}}{dt}= \alpha {e}^{-\updelta {Q}_{t}}$$

The adsorption isotherms provide information on the distribution of adsorbate (Diclofenac) in the solid and liquid phase after attaining equilibrium. The Langmuir adsorption model assumes that saturation monolayer adsorption takes place on the adsorbent’s surface with no interaction between the adsorbed molecules. It aids to explain the direct adsorption from aqueous medium^32^. The linear form of Langmuir model is expressed by the Eq. (7), where equilibrium concentration C_e_ (mg L^−1^), amount adsorbed at equilibrium Q_e_ (mg g^−1^) and Langmuir constants (X_m_ and K_L_) related to the adsorption efficiency and energy, respectively. The Langmuir model’s assumption is that the surface adsorption takes place at homogeneous sites of POHC and the favorability of the process is confirmed by the separation factor, R_L_ which is expressed in Eq. (8). The Freundlich isotherm postulate a non-uniform distribution of heterogeneous sites and do not assumes a monolayer adsorption capacity. It can be given by the Eq. (9), with the constants affecting the adsorption capacity k_f_ and n. The log Q_e_ versus C_e_ plot suggests the Freundlich isotherm nature. If the value of n ranges from 1 to 10, then the adsorption is considered to be feasible and good specifically, the values found more than 1 indicates its stronger adsorption force on the active sites of the hydrochar^33^. The linear form of Temkin isotherm model is expressed by Eq. (10). The constants of Temkin isotherm i.e., equilibrium binding constant, K_t_ (L g^−1^) and constant related to heat of absorption, B (KJ mol^−1^) are calculated from the intercept and slope of Q_e_ versus ln C_e_ plot with the universal gas constant, R (0.008314 kJ mol^−1^) and the absolute temperature (T) in K^34^.

7 $$\frac{{c}_{e}}{{q}_{e}}=\frac{1}{{{K}_{L}X}_{m}}+\frac{{c}_{e}}{{X}_{m}}$$

8 $${\text{R}}_{{\text{L}}} = {1}/{1} + {\text{X}}_{{\text{m}}} {\text{K}}_{{\text{L}}}$$

9 $$\text{log}{ q}_{e}=\text{log}{ k}_{f}+ \frac{1}{n}\text{log}{C}_{e}$$

10 $${q}_{e}= \frac{RT}{B}\text{ln}{K}_{T}+ \frac{RT}{B}\text{ln}{C}_{e}$$

Diclofenac sodium^35^ (Table S1) was purchased from M/s Sigma Aldrich (Merck). Type I and Type III water were used from Merck-Millipore unit for all the procedures with a resistivity of 18 MΩ cm. After each adsorption experiment, centrifugation was performed at 6000 rpm for 10 min followed by subsequent filtration. The measurement of Diclofenac was carried out at 276 nm using a Shimadzu UV-1800 UV–Vis spectrophotometer^36^.

## Results and discussion

### Hydrochar characterization

#### Structural morphologies and textural characterization of hydrochars

**Figure 2:**
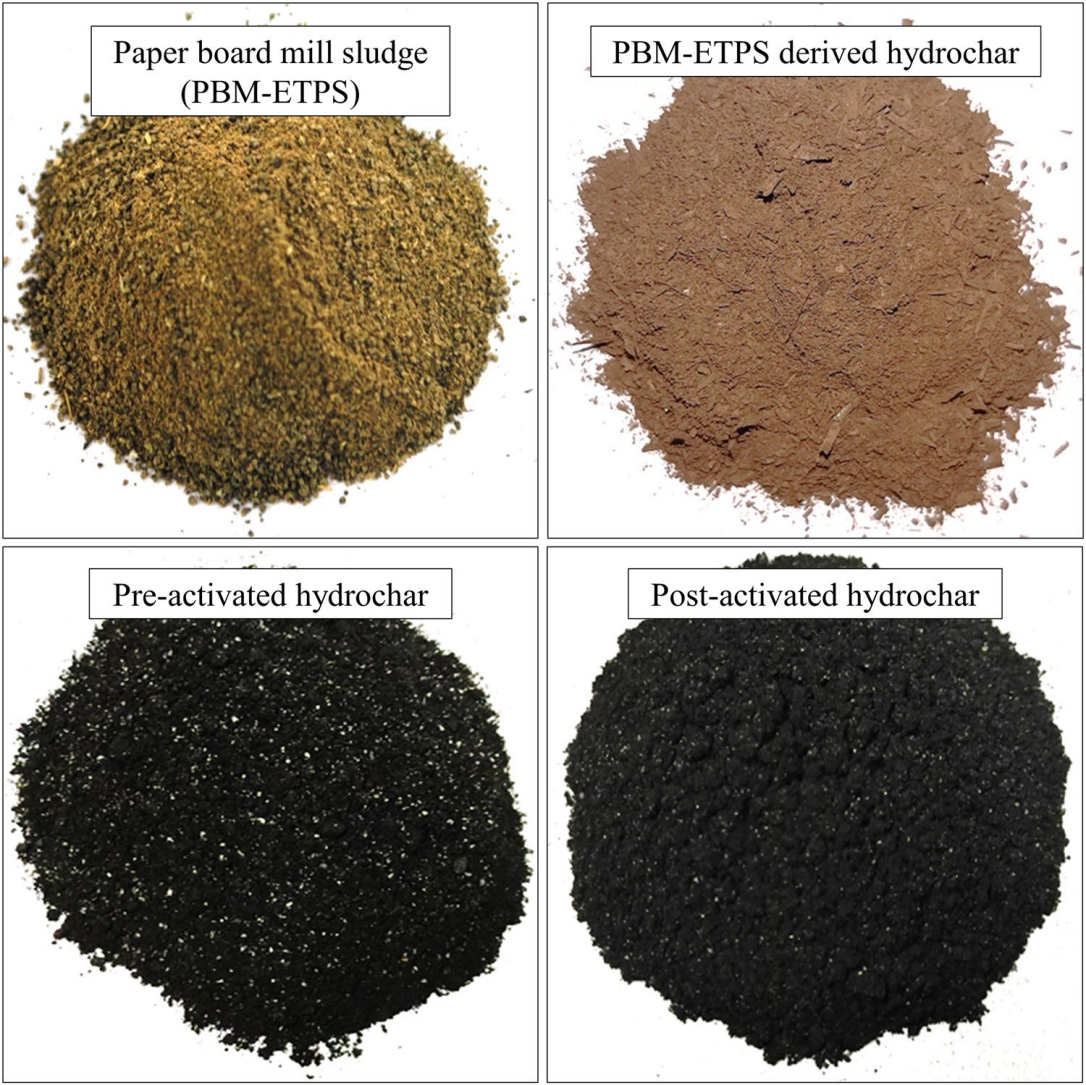
Visual interpretation of Paper board mill ETP sludge and its derived hydrochars.

**Figure 3:**
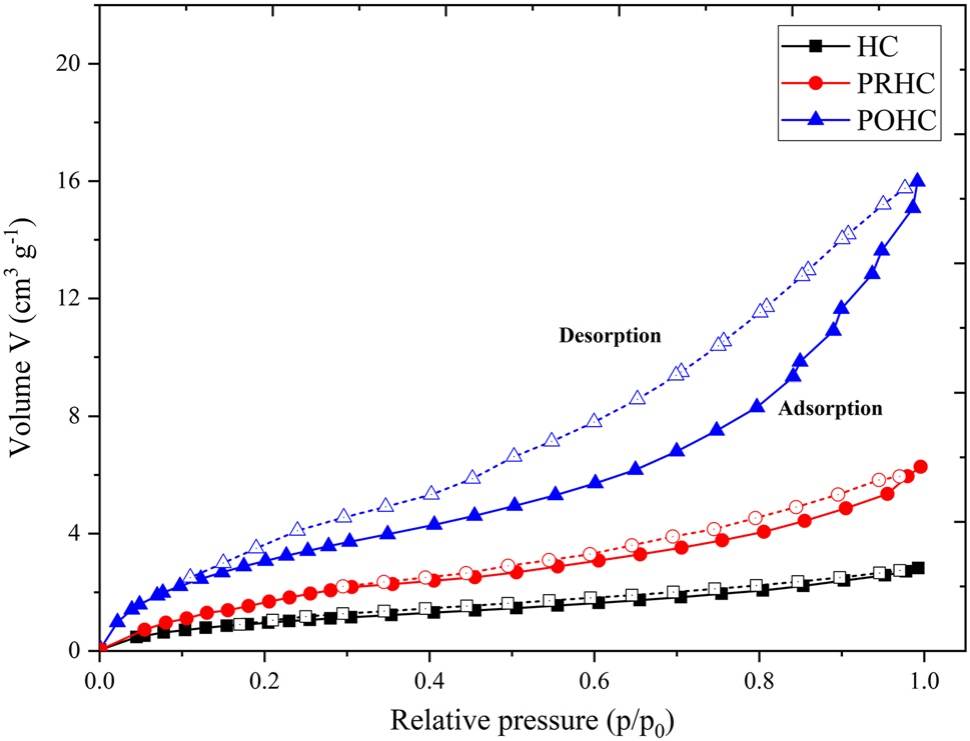
The N_2_ adsorption/desorption isotherm.

**Figure 4:**
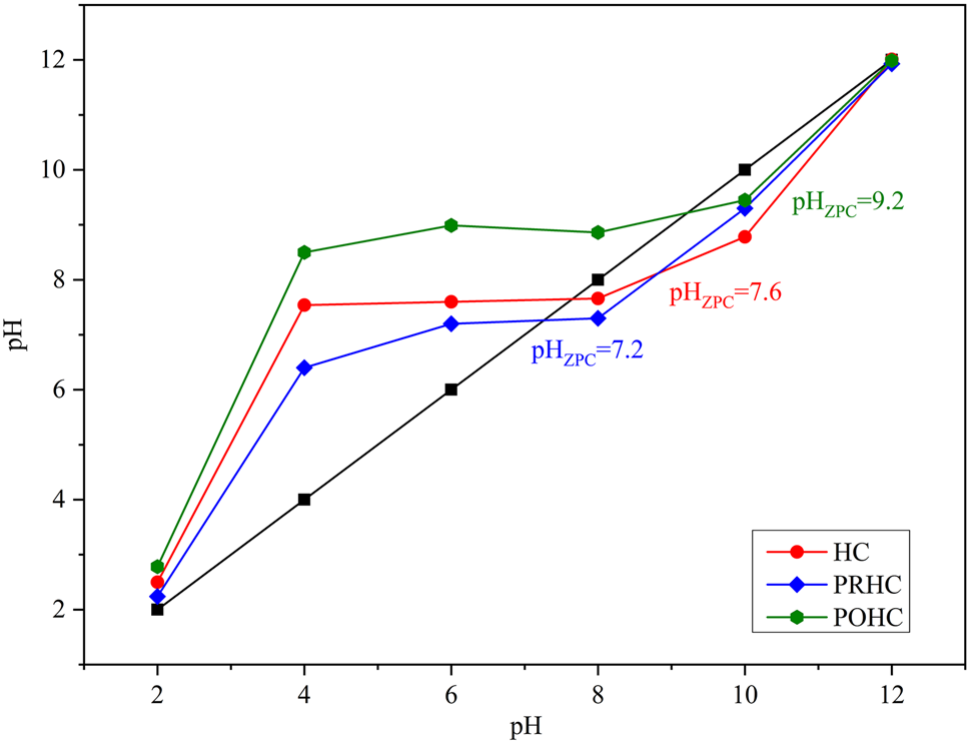
Point of Zero charge for hydrochars.

The color and texture of hydrochars visually varied with each other wherein the activated hydrochars depicted a dark fine texture (Fig. 2). The BET surface area were 19.59 m^2^g^−1^, 31.08 m^2^g^−1^ and 53.32 m^2^g^−1^ for HC, PRHC and POHC, respectively. The data obtained from N_2_ adsorption/desorption were used to plot the isotherm graphs (Fig. 3). All the hydrochars (HC, PRHC and POHC) correlated with type III isotherm (convex to p/p_0_ axis), which indicates weak adsorbent-adsorbate interactions with lower heat of adsorption than the heat of liquification^37^. According to BET theory, the energy of monolayer adsorption is exponentially related to the parameter C. The value of “C” (intercept) in the BET plot plays a key role in deciding the type of isotherm. Although the adsorption hysteresis occurs in the reported graph, the C value in the constructed BET plot was less than 2, thereby indicating the isotherm type to be type III^37^. On the surface of the adsorbent, the adsorbed molecules clusters around the most favorable sites and their amount stays finite at the saturation pressure. However, the adsorption proceeds due to greater interaction of adsorbate with an already adsorbed layer than its interaction with the adsorbent surface^38^. The zeta potential of the HC was − 17.1 mV while, it further reduced to − 20.6 mV and − 38 mV for PRHC and POHC, respectively. The surface charge was enhanced due to the existence of oxygenated functional groups that led to the higher adsorption capacities^39^. The cation exchange capacity of the HC was 12 c.mol [p^+^] kg^−1^ and it surged to 14 c.mol [p^+^] kg^−1^ and 20 c.mol [p^+^] kg^−1^ for PRHC and POHC, respectively. The pH_zpc_ were 7.6, 7.2 and 9.2 for HC, PRHC and POHC, respectively (Fig. 4). The point of zero charge indicates the pH at which the carbon material acquires nil charge on its surface and it helps to detect the type of charge present on the adsorbent surface at the solution pH. The hydrochar gained positive charge at the solution pH below pH_zpc_, thus it is highly favourable for the adsorption of cationic contaminants. The acid dissociation constant (p*K*_a_) of Diclofenac vary substantially depending on its molecular structure. Diclofenac is transformed to an anion or cation depending on their p*K*_a_ (~ 4.15) and solution pH, thus pH alterations can significantly affect the Diclofenac adsorption. When the pH of the solution is less than p*K*_a_, Diclofenac exists in its neutral (undissociated) state^40^. Diclofenac, on the other hand, assumes its anionic (dissociation) form at pH > p*K*_a_ and a positively charged surface of hydrochar; HC, POHC and PRHC existed until pH ~ 7.6, 7.2 and 9.2, respectively^40^.

**Figure 5:**
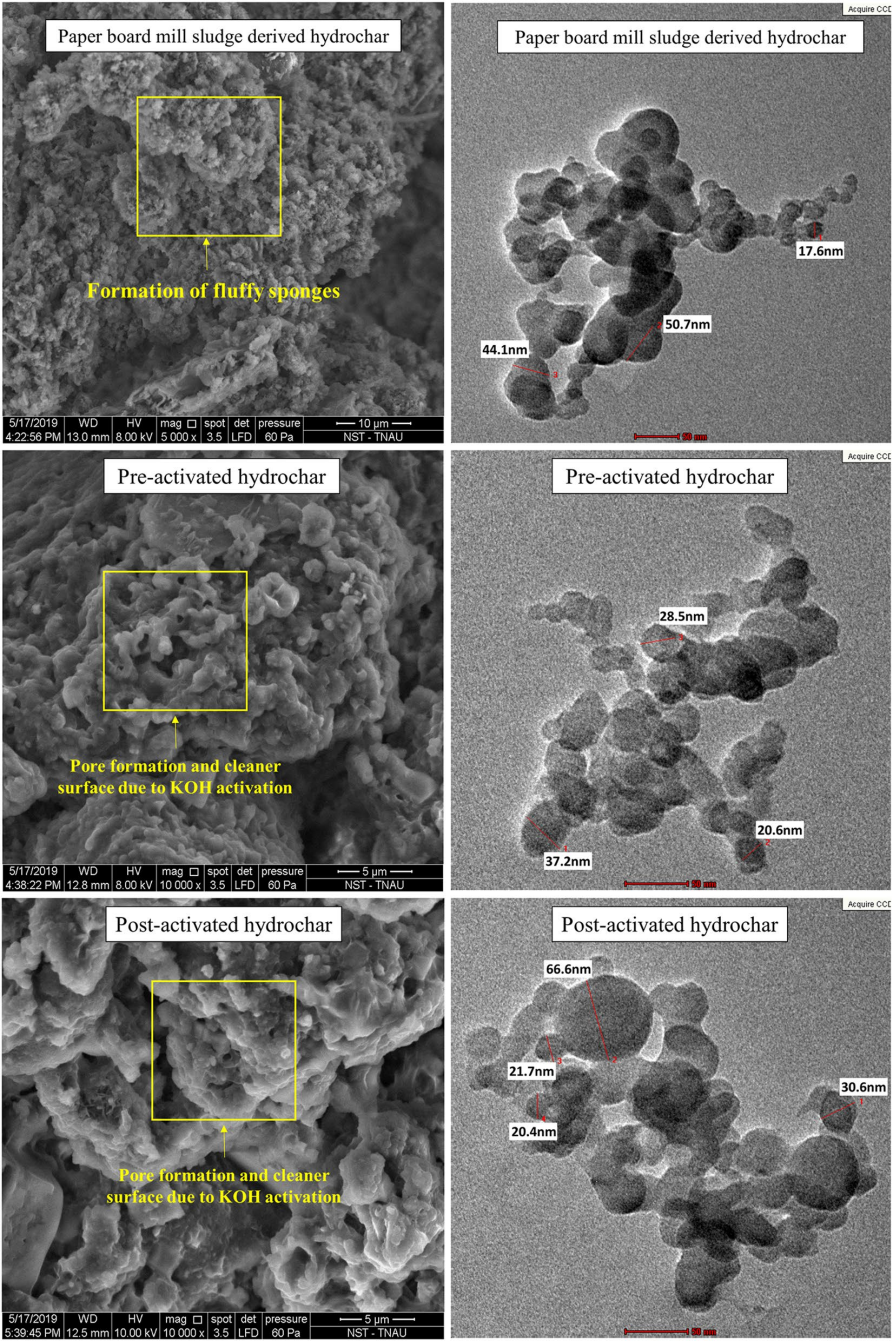
SEM and TEM micrographs of hydrochars.

The SEM micrographs of hydrochars (Fig. 5) exhibited the formation of microspheres and pores due to intensive carbonization during KOH activation. This helps in the enhancement of hydrochar adsorption potential for various environmental contaminants. The TEM micrographs of hydrochars (Fig. 5) also confirmed the formation of porous spherical shaped carbon nanoparticles. The activation produced carbon nanospheres ranging between 20 and 60 nm. Additionally, the KOH promoted the impurity removal from cracks and pores of activated hydrochars thus making it as a cleaner surface. Similar findings were reported in the hydrothermal carbonization of wheat straw, corn stalk and saw dust wherein the KOH activation produced porous carbon with cleaner surface and promoted heavy metal removal from wastewater^39^.

#### Molecular functionalities of hydrochars

**Figure 6:**
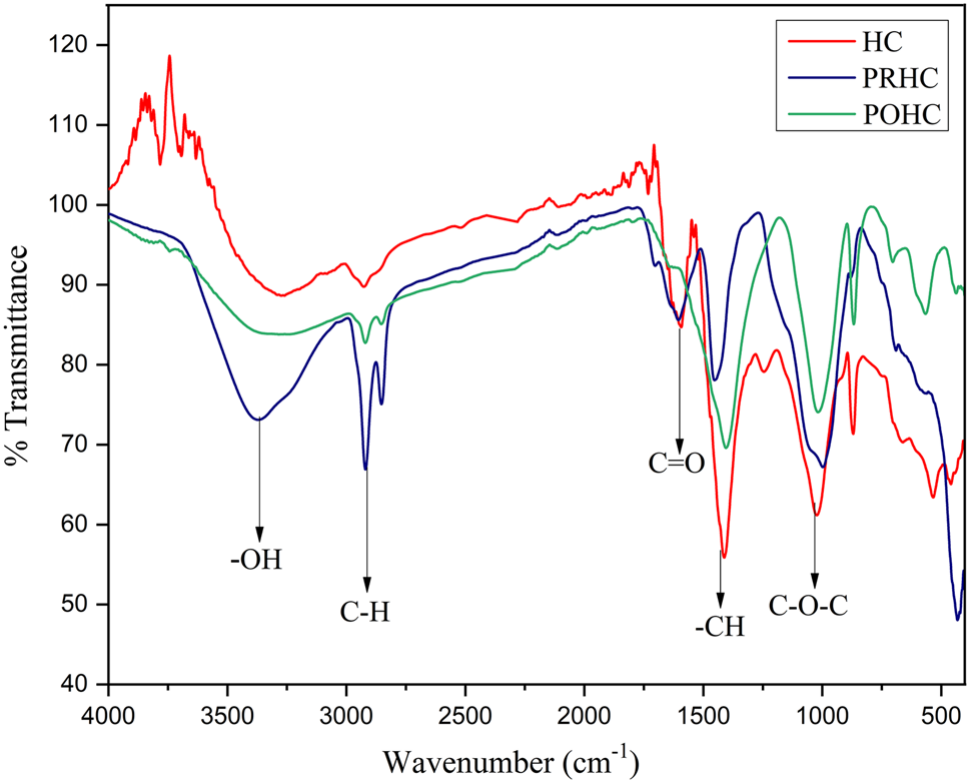
FTIR spectra of hydrochars.

The FTIR spectra of hydrochars (Fig. 6) exhibited a variable broad OH stretching band around 3200–3600 cm^−1^ with a blunted tip at 3260 cm^−1^ due to the presence of phenolic OH groups and cellulose, respectively. The aliphatic methyl group’s vibration were represented by the band around 2800–3000 cm^−1^ with a centroid at 2920 cm^−1^ that exhibited the amino acids presence due to asymmetric C–H stretching. The amide group formed proteins were depicted by the stretching band of C=O at 1592 cm^−1^. The presence of –CH in the form of –CH_2_ and –CH_3_ was depicted by a sharp peak at 1412 cm^−1^. The dehydration of alcohol was represented by the asymmetric stretching of C–O–C at 1026 cm^−1^. The activation of hydrochar increased stretching of O–H, C–H, aromatic C=C, aromatic C=O and C–H bands. The OH deprotonating of activated hydrochar was noticed due to lower C–O–C vibration at 1026 cm^−1^^8^. The KOH activation further boosted up the formation of oxygenated functional groups especially the carboxylic groups that caused the lower zeta potential values^39^. These oxygenated functional groups influenced and further reduced the zeta potential after activation from − 17.1 mV (HC) to − 20.6 mV and − 38 mV for PRHC and POHC, respectively.

### Adsorption of diclofenac by hydrochars

#### Adsorption kinetics

**Figure 7:**
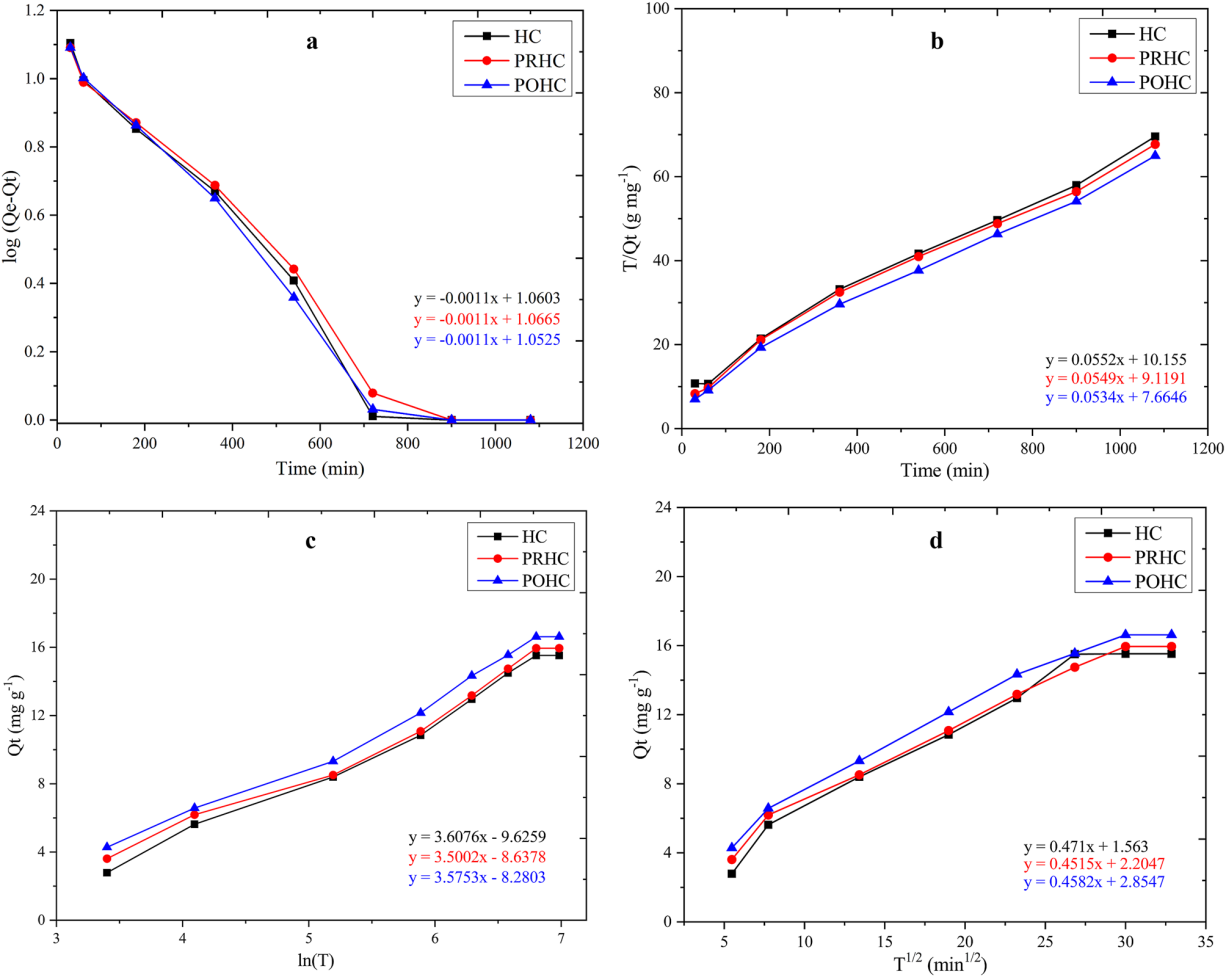
Kinetics of Diclofenac removal (**a**) Pseudo-first order, (**b**) Pseudo-second order, (**c**) Elovich and (**d**) Intraparticle diffusion.

The kinetic models employed for the Diclofenac adsorption by hydrochars were first-order reaction, second order reaction, Elovich model and intra particle diffusion model (Table 1). The model equations are commonly used to assess different adsorbent materials on the basis of rate constants at any given time interval. The experimental kinetic data’s goodness of fit were tested by co-efficient of determination (R^2^). The intercept and slope value of the log (Q_e_ − Q_t_) versus time plot resolved rate constant (k_1_) for pseudo-first order kinetic model (Fig. 7a). The experimental results showed better fit for the pseudo second order kinetics. The maximum value of R^2^ was obtained for second-order kinetics reaction in case of HC (0.9918), PRHC (0.9876) and POHC (0.9924) indicating the fitness of adsorption to second-order kinetics (Fig. 7b). The rate of adsorption was faster within 12 h of adsorption time, indicating a wide range of pores on the surface of HC, POHC, and PRHC. The process attains equilibrium when q_t_ values reached constant value after 15 h. Diclofenac's ionic form can be ion-exchanged with hydroxyl or carboxyl groups on the sludge or connected in the form of conjugated electron pairs^40,41^.

**Table 1:** Kinetic parameters for Diclofenac adsorption by hydrochars.

	HC	PRHC	POHC
**Pseudo first order**			
R^2^	0.9402	0.9577	0.9434
K_1_ (min^−1^)	0.0025	0.0025	0.0025
**Pseudo second order**			
R^2^	0.9918	0.9876	0.9924
K_2_ (g mg^−1^ min^−1^)	32.317	36.383	45.753
**Elovich model**			
R^2^	0.988	0.98	0.987
α (mg g^−1^ min^−1^)	3.2522	2.3717	2.2101
β (mg g^−1^)	0.2771	0.2856	0.2796
**Intra particle diffusion**			
R^2^	0.96	0.979	0.973
K_i_ (mg g^−1^ h^1/2^)	0.471	0.451	0.458

The major forces to be responsible for the adsorption of Diclofenac on the adsorbent surface include physisorption or chemisorption^32^. Electrostatic interactions between Diclofenac and positively charged surface of HC, POHC and PRHC are the responsible driving forces for Diclofenac adsorption. In addition, interactions such as hydrophobic effects, van der Waals forces, and interactions play a role in the adsorption of Diclofenac onto hydrochar^42^. The results obtained from the experiment revealed that the lowest adsorption capacity was found for HC (15.53 mg g^−1^) than PRHC (15.95 mg g^−1^) and POHC (16.63 mg g^−1^). The KOH activated hydrochar exhibited higher adsorption capacity of Diclofenac due to the reasons such as higher proportion of oxygenated functional groups, higher pH_zpc_ values and negative zeta potential with slightly higher surface area than pristine hydrochar^25^. Moreover, the adsorption of Diclofenac depends on both concentration and time^33^. The coefficient of determination of the Elovich (Fig. 7c) and intraparticle diffusion (Fig. 7d) models were lower compared to pseudo first and second order models^43^. The results were found contradictory with the results of adsorption of pharmaceutical drugs on the adsorbents like graphene oxide and graphene^44^, coal based activated charcoal^45^ and activated carbon^46^. However, similar trend was reported in the removal of Diclofenac, salicylic acid and flubiprofen from KOH activated orange peel hydrochar which obeyed pseudo-second order adsorption kinetic model^11^.

#### Adsorption isotherm

**Figure 8:**
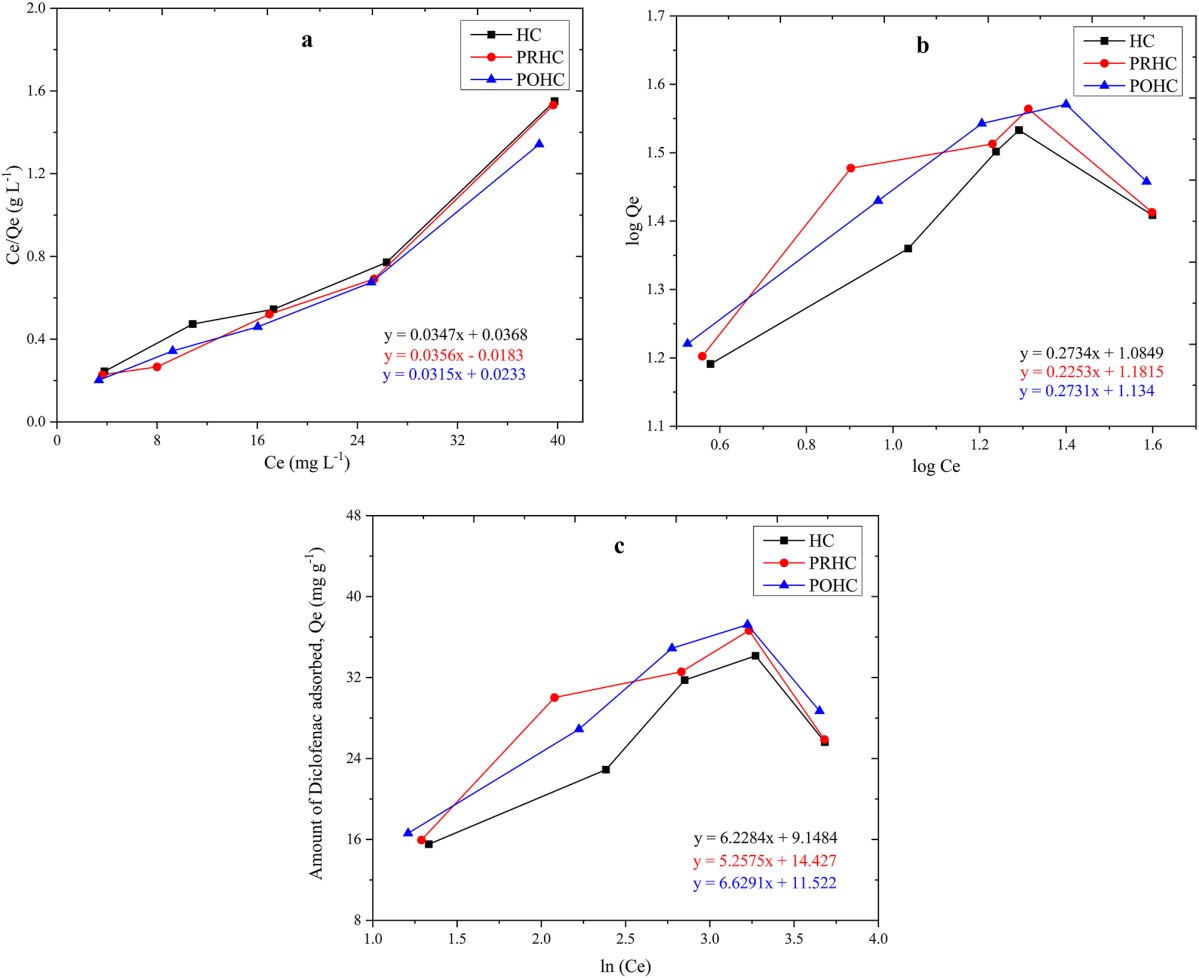
Isotherms of Diclofenac removal (**a**) Langmuir, (**b**) Freundlich and (**c**) Temkin.

The adsorption capacities of different hydrochars at varying initial equilibrium concentration of Diclofenac were assessed using linear Freundlich, Langmuir and Temkin isotherm models (Table 2). The behavior of hydrochars (HC, POHC, and PRHC) showed similar trend towards Diclofenac adsorption at an equilibrium time of 15 h (Fig. 8). The isothermal adsorption curves of Diclofenac for HC (R^2^-0.932), PRHC (R^2^-0.934) and POHC (R^2^-0.948) showed that the three hydrochars conformed Langmuir model, indicating the mechanism to be mono-layered (Fig. 8a). The values obtained for R_L_ ranged from 0 to 1, indicating that the adsorption process was favorable for HC (0.96), PRHC (0.98) and POHC (0.97)^32^. The R^2^ value of Freundlich isotherm (Fig. 8b) for HC (0.58), PRHC (0.41) and POHC (0.66) were lower than the corresponding values of Langmuir isotherm suggesting its non-applicability in the adsorption of Diclofenac. The Temkin model (Fig. 8c) became less appropriate for the Diclofenac adsorption on the hydrochars due to much lower R^2^ values.

**Table 2:** Isotherm parameters for Diclofenac adsorption by hydrochars.

	HC	PRHC	POHC
**Langmuir**			
R^2^	0.932	0.934	0.948
X_m_ (mg g^−1^)	28.818	28.09	31.746
K_l_ (L g^−1^)	0.0012	0.0006	0.0007
R_L_	0.964	0.982	0.977
**Freundlich**			
R^2^	0.58	0.41	0.665
K_f_ (mg^1–1/n ^L^1/n^ g^−1^)	2.959	3.259	3.108
n	3.658	4.439	3.662
**Temkin**			
R^2^	0.582	0.4	0.608
B (KJ mol^−1^)	0.398	0.471	0.374
K_t_ (L g^−1^)	4.344	15.55	5.687

**Figure 9:**
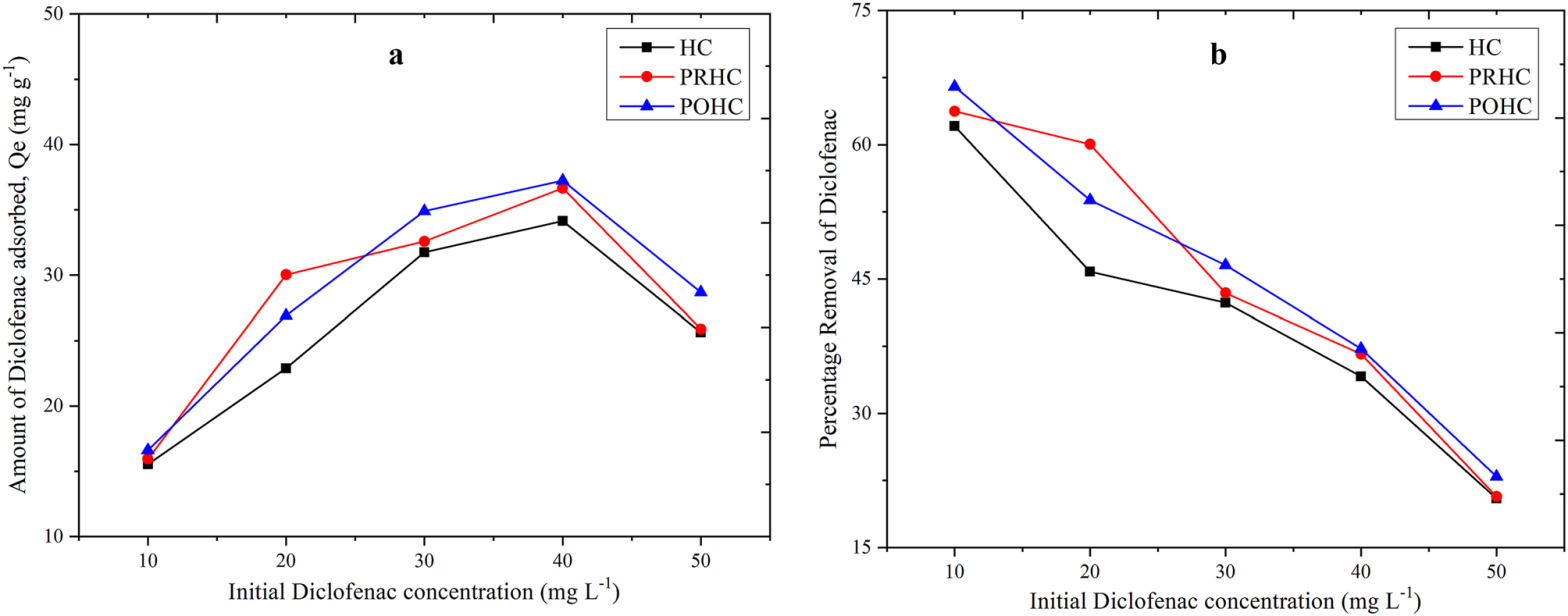
Diclofenac removal at equilibrium time.

The adsorption capacities of POHC (37.23 mg g^−1^), PRHC (36.65 mg g^−1^) and HC (34.15 mg g^−1^) at an initial Diclofenac concentration of 40 mg L^−1^ and 15 h equilibrium period were found to be very close (Fig. 9a). At the beginning, obvious adsorption occurred within 12 h by all of the adsorbents. As the adsorption time increased further, there was an increase in adsorption capacity up to 15 h and a dip was obtained in all the adsorbents thereby indicating the equilibrium time to be 15 h. Since the adsorbents were derived from paper board mill sludge with moderately low carbon content and their surface area were observed to have a little difference, very close adsorption capacities were observed^41^. The pre-activation and post-activation of hydrochars yielded significant effects on the generation of oxygenated functional groups, yet their Diclofenac adsorption capacities were not as high as some commercial activated carbon due to lower carbon content and surface area^41^. This suggests the direct utilization of pristine sludge derived hydrochar without activation for the removal of emerging contaminants thereby confirming its cost-effectiveness. However, the removal efficiency of Diclofenac decreased gradually with corresponding increase in initial Diclofenac concentration due to the filling up of pore space (Fig. 9b). A comparison of the present study with the related previous studies involving the adsorption of Diclofenac is illustrated in the Table S2^47–56^. It can be seen that sludge derived hydrochar exhibited higher adsorption capacity towards Diclofenac than other carbon materials produced from pine bark, palm kernel shell and orange peel.

The yield of hydrochar produced from paper board mill sludge was determined in our previous study as 70%^1^. The yield after activation decreased for the PRHC and POHC to 59% and 65%, respectively. Production and commercial practicability of hydrochar is essential in the mass production. Since HC, POHC and POHC had similar adsorption capacities, the use of HC can be a noble choice for the removal of the emerging contaminants from wastewater. The results of this investigation showed that the synthesis of hydrochar from paper board mill sludge is a simple process that does not require any pre-treatment process. Activated carbon in comparison with hydrochar production involves a very high temperature and expensive chemicals. More notably, hydrochar has reported to have a higher adsorption capacity for pharmaceuticals adsorption than other adsorbents (Table S2). Furthermore, the use of hydrochar (HC, PRHC, and POHC) to remove Diclofenac from water is a viable option under a variety of environmental conditions, including pH and high pollutant concentrations (up to 10–50 mg L^−1^ in this study).

## Conclusion

The activation of hydrochar yielded carbon micro-spheres with higher fraction of oxygenated functional groups and facilitated Diclofenac removal from aqueous solution. Hence, the risk of emerging contaminants in food chain can be controlled. This novel approach promotes simultaneous management of solid waste (sludge) and serves as water treatment option in promoting environmental sustainability. Future research will focus on the removal of emerging contaminants from natural water bodies as a scaled up study in this study area. Furthermore, new activated carbons can be produced from hydrochars for the enhancement of their adsorption capacities and development of novel applications.

## Supplementary Information

Supplementary Tables.
