# Supplementary material for: Kinetic and isotherm insights of Diclofenac removal by sludge derived hydrochar
Source: Sci Rep. 2022 Feb 9;12:2184. doi: 10.1038/s41598-022-05943-z (PMC8828768; doi:10.1038/s41598-022-05943-z)
Supplement: Supplementary file 1 — Supplementary Tables. [file 41598_2022_5943_MOESM1_ESM.docx]

**Supplementary information**

**Table S1. Description of Diclofenac sodium salt**

| Molecular formula | C_14_H_10_Cl_2_NNaO_2_ |
| --- | --- |
| Chemical Name | 2-[(2,6-dichlorophenyl)amino] benezene acetic acid sodium salt |
| Molecular weight | 318.13 g mol^-1^ |
| Wavelength of maximum absorption | 276 nm |
| Solubility in water | 50 mg ml^-1^ |
| pK_a_ | 4.15 |

**Table S2. Comparison of diclofenac adsorption by different types of carbon material**

| Adsorbent | Activating agent | Adsorption conditions | Surface area (m^2^ g^-1^) | Maximum adsorption capacity (mg g^-1^) | References |
| --- | --- | --- | --- | --- | --- |
| Sludge derived hydrochar | - | C_o­_= 10 to 50 mg L^-1^  pH- neutral  Time – 15 h | 19.59 | 34.15 | This work |
| PRHC | KOH pre-activation |  | 31.08 | 36.65 |  |
| POHC | KOH post-activation |  | 53.32 | 37.23 |  |
| Palm kernel shell hydrochar | Air | C_o_- 0 to 50 mg L^-1^  pH - 2  Time – 1.5 h | 131 | 13.16 | ^49^ |
| Pristine polypyrrole | - | pH – 6  Time – NA | NA | 223 | ^50^ |
| Thermo-plasma expanded graphite | - | C_o_ – 10 to 250 mg L^-1^  pH - 1  Time – 22 h | 47 | 433.29 | ^51^ |
| Activated carbon | FeCl_3_ | C_o_ – 10 to 200 mg L^-1^  pH – 4.5  Time – 24 h | 457 | 144 | ^52^ |
| Activated carbon | K_2_CO_3_ |  | 184 | 5.61 |  |
| Orange peel hydrochar | - |  | 3.56 | 6.44 |  |
| Carbon from tea waste | ZnCl_2_ | C_o_ – 10 to 50 mg L^-1^  pH – 6.5  Time – 6 h | 865 | 62 | ^53^ |
| Synthetic porous carbon | ZnCl_2_ | C_o_ – 50 mg L^-1^  pH – 6.5  Time – 3 h | 1224 | 309 | ^54^ |
| Graphene oxide | - | C_o_ – 400 mg L^-1^  pH – 6  Time – 14.75 min | NA | 653.91 | ^55^ |
| Pine Bark | NA | C_o_ – 50 to 400 mg L^-1^  pH – 7  Time – 0.75 h | NA | 54.7 | ^56^ |

**Table S3. Kinetics of Diclofenac adsorption by Hydrochar (HC)**

| Time (min) | Initial Diclofenac concentration (mg L^-1^) Co | Final Diclofenac concentration (mg L^-1^) Ce | Amount removed  (mg L^-1^) | Percent removal | Amount removed (mg g^-1^) Qe | Qe-Qt (mg g^-1^) | log  (Qe-Qt) | T^1/2^ min^1/2^ | ln T | T/Q_t_  (g mg^-1^) | ln Qt |
| --- | --- | --- | --- | --- | --- | --- | --- | --- | --- | --- | --- |
| 30 | 10 | 8.88 | 1.12 | 11.16 | 2.79 | 12.74 | 1.10 | 5.48 | 3.40 | 10.75 | 1.03 |
| 60 | 10 | 7.75 | 2.25 | 22.51 | 5.63 | 9.90 | 1.00 | 7.75 | 4.09 | 10.66 | 1.73 |
| 180 | 10 | 6.64 | 3.36 | 33.59 | 8.40 | 7.13 | 0.85 | 13.42 | 5.19 | 21.43 | 2.13 |
| 360 | 10 | 5.66 | 4.34 | 43.38 | 10.85 | 4.68 | 0.67 | 18.97 | 5.89 | 33.20 | 2.38 |
| 540 | 10 | 4.82 | 5.19 | 51.85 | 12.96 | 2.56 | 0.41 | 23.24 | 6.29 | 41.66 | 2.56 |
| 720 | 10 | 4.20 | 5.80 | 58.00 | 14.50 | 1.03 | 0.01 | 26.83 | 6.58 | 49.66 | 2.67 |
| 900 | 10 | 3.79 | 6.21 | 62.10 | 15.53 | 0.00 | 0.00 | 30.00 | 6.80 | 57.97 | 2.74 |
| 1080 | 10 | 3.79 | 6.21 | 62.10 | 15.53 | 0.00 | 0.00 | 32.86 | 6.98 | 69.57 | 2.74 |

**Table S4. Kinetics of Diclofenac adsorption by Pre-activated Hydrochar (PRHC)**

| Time (min) | Initial Diclofenac concentration (mg L^-1^) Co | Final Diclofenac concentration (mg L^-1^) Ce | Amount removed  (mg L^-1^) | Percent removal | Amount removed (mg g^-1^) Qe | Qe-Qt (mg g^-1^) | log  (Qe-Qt) | T^1/2^ min^1/2^ | ln T | T/Q_t_  (g mg^-1^) | ln Qt |
| --- | --- | --- | --- | --- | --- | --- | --- | --- | --- | --- | --- |
| 30 | 10 | 8.56 | 1.44 | 14.44 | 3.61 | 12.34 | 1.09 | 5.48 | 3.40 | 8.31 | 1.28 |
| 60 | 10 | 7.52 | 2.48 | 24.76 | 6.19 | 9.76 | 0.99 | 7.75 | 4.09 | 9.69 | 1.82 |
| 180 | 10 | 6.59 | 3.41 | 34.06 | 8.52 | 7.44 | 0.87 | 13.42 | 5.19 | 21.14 | 2.14 |
| 360 | 10 | 5.57 | 4.43 | 44.31 | 11.08 | 4.87 | 0.69 | 18.97 | 5.89 | 32.50 | 2.40 |
| 540 | 10 | 4.73 | 5.27 | 52.72 | 13.18 | 2.77 | 0.44 | 23.24 | 6.29 | 40.97 | 2.58 |
| 720 | 10 | 4.10 | 5.90 | 59.00 | 14.75 | 1.20 | 0.08 | 26.83 | 6.58 | 48.81 | 2.69 |
| 900 | 10 | 3.62 | 6.38 | 63.80 | 15.95 | 0.00 | 0.00 | 30.00 | 6.80 | 56.43 | 2.77 |
| 1080 | 10 | 3.62 | 6.38 | 63.80 | 15.95 | 0.00 | 0.00 | 32.86 | 6.98 | 67.71 | 2.77 |

**Table S5. Kinetics of Diclofenac adsorption by Post-activated Hydrochar (POHC)**

| Time (min) | Initial Diclofenac concentration (mg L^-1^) Co | Final Diclofenac concentration (mg L^-1^) Ce | Amount removed  (mg L^-1^) | Percent removal | Amount removed (mg g^-1^) Qe | Qe-Qt (mg g^-1^) | log  (Qe-Qt) | T^1/2^ min^1/2^ | ln T | T/Q_t_  (g mg^-1^) | ln Qt |
| --- | --- | --- | --- | --- | --- | --- | --- | --- | --- | --- | --- |
| 30 | 10 | 8.29 | 1.71 | 17.13 | 4.28 | 12.34 | 1.09 | 5.48 | 3.40 | 7.01 | 1.45 |
| 60 | 10 | 7.37 | 2.63 | 26.31 | 6.58 | 10.05 | 1.00 | 7.75 | 4.09 | 9.12 | 1.88 |
| 180 | 10 | 6.27 | 3.73 | 37.29 | 9.32 | 7.30 | 0.86 | 13.42 | 5.19 | 19.31 | 2.23 |
| 360 | 10 | 5.14 | 4.86 | 48.63 | 12.16 | 4.47 | 0.65 | 18.97 | 5.89 | 29.61 | 2.50 |
| 540 | 10 | 4.27 | 5.74 | 57.35 | 14.34 | 2.29 | 0.36 | 23.24 | 6.29 | 37.66 | 2.66 |
| 720 | 10 | 3.78 | 6.22 | 62.20 | 15.55 | 1.08 | 0.03 | 26.83 | 6.58 | 46.30 | 2.74 |
| 900 | 10 | 3.35 | 6.65 | 66.50 | 16.63 | 0.00 | 0.00 | 30.00 | 6.80 | 54.14 | 2.81 |
| 1080 | 10 | 3.35 | 6.65 | 66.50 | 16.63 | 0.00 | 0.00 | 32.86 | 6.98 | 64.96 | 2.81 |

**Table S6. Isotherm of Diclofenac adsorption by Hydrochar (HC) at equilibrium time**

| Initial Diclofenac concentration (mg L^-1^) Co | Final Diclofenac concentration (mg L^-1^) Ce | Amount removed  (mg L^-1^) | Amount removed (mg g^-1^) Qe | Percent removal | Ce/Qe, (g L^-1^) | 1/Qe  (g mg^-1^) | Qe/Ce | log Ce | log Qe | ln Ce |
| --- | --- | --- | --- | --- | --- | --- | --- | --- | --- | --- |
| 10 | 3.79 | 6.21 | 15.53 | 62.10 | 0.24 | 0.06 | 4.10 | 0.58 | 1.19 | 1.33 |
| 20 | 10.84 | 9.16 | 22.90 | 45.80 | 0.47 | 0.04 | 2.11 | 1.04 | 1.36 | 2.38 |
| 30 | 17.30 | 12.70 | 31.75 | 42.33 | 0.54 | 0.03 | 1.84 | 1.24 | 1.50 | 2.85 |
| 40 | 26.34 | 13.66 | 34.15 | 34.15 | 0.77 | 0.03 | 1.30 | 1.42 | 1.53 | 3.27 |
| 50 | 39.75 | 10.25 | 25.63 | 20.50 | 1.55 | 0.04 | 0.64 | 1.60 | 1.41 | 3.68 |

**Table S7. Isotherm of Diclofenac adsorption by Pre-activated Hydrochar (PRHC) at equilibrium time**

| Initial Diclofenac concentration (mg L^-1^) Co | Final Diclofenac concentration (mg L^-1^) Ce | Amount removed  (mg L^-1^) | Amount removed (mg g^-1^) Qe | Percent removal | Ce/Qe, (g L^-1^) | 1/Qe  (g mg^-1^) | Qe/Ce | log Ce | log Qe | ln Ce |
| --- | --- | --- | --- | --- | --- | --- | --- | --- | --- | --- |
| 10 | 3.63 | 6.374 | 15.94 | 63.74 | 0.23 | 0.06 | 4.39 | 0.56 | 1.20 | 1.28 |
| 20 | 7.99 | 12.01 | 30.03 | 60.05 | 0.27 | 0.03 | 3.76 | 0.90 | 1.48 | 2.07 |
| 30 | 16.97 | 13.03 | 32.58 | 43.43 | 0.52 | 0.03 | 1.92 | 1.23 | 1.51 | 2.83 |
| 40 | 25.34 | 14.66 | 36.65 | 36.65 | 0.69 | 0.03 | 1.45 | 1.40 | 1.56 | 3.23 |
| 50 | 39.65 | 10.35 | 25.88 | 20.70 | 1.53 | 0.04 | 0.65 | 1.60 | 1.41 | 3.68 |

**Table S8. Isotherm of Diclofenac adsorption by Post-activated Hydrochar (POHC) at equilibrium time**

| Initial Diclofenac concentration (mg L^-1^) Co | Final Diclofenac concentration (mg L^-1^) Ce | Amount removed  (mg L^-1^) | Amount removed (mg g^-1^) Qe | Percent removal | Ce/Qe, (g L^-1^) | 1/Qe  (g mg^-1^) | Qe/Ce | log Ce | log Qe | ln Ce |
| --- | --- | --- | --- | --- | --- | --- | --- | --- | --- | --- |
| 10 | 3.35 | 6.65 | 16.62 | 66.48 | 0.20 | 0.06 | 4.96 | 0.53 | 1.22 | 1.20 |
| 20 | 9.24 | 10.76 | 26.90 | 53.80 | 0.34 | 0.04 | 2.91 | 0.97 | 1.43 | 2.22 |
| 30 | 16.04 | 13.96 | 34.90 | 46.53 | 0.46 | 0.03 | 2.18 | 1.21 | 1.54 | 2.77 |
| 40 | 25.11 | 14.89 | 37.23 | 37.23 | 0.67 | 0.03 | 1.48 | 1.40 | 1.57 | 3.22 |
| 50 | 38.52 | 11.48 | 28.69 | 22.95 | 1.34 | 0.03 | 0.74 | 1.59 | 1.46 | 3.65 |
